# Measuring emotional intelligence with the MSCEIT 2: theory, rationale, and initial findings

**DOI:** 10.3389/fpsyg.2025.1539785

**Published:** 2025-06-02

**Authors:** John D. Mayer, David R. Caruso, Peter Salovey, Iris Y. Lin, Braden J. Hansma, Joanna Solomon, Gill Sitarenios, Manolo Romero Escobar

**Affiliations:** ^1^Department of Psychology, University of New Hampshire, Durham, NH, United States; ^2^Office of the Dean of Yale College, Yale University, New Haven, CT, United States; ^3^Department of Psychology, Yale University, New Haven, CT, United States; ^4^Research and Development, Multi-Health Systems, Inc., Toronto, ON, Canada

**Keywords:** emotional intelligence, assessment, four-domain model, CHC Model, factor analysis, veridical scoring, Mayer-Salovey-Caruso Emotional Intelligence Test 2 (MSCEIT 2)

## Abstract

**Introduction:**

The model of emotional intelligence as an ability has evolved since its introduction 35 years ago. The revised model includes that emotional intelligence (EI) is a broad intelligence within the Cattell-Horn-Carroll (CHC) model of intelligence, and that more areas of problem solving are involved than originally detailed. An argument is made here that *veridical* scoring of EI test responses is a sound procedure relative to scoring keys based on expert consensus or a single emotion theory. To the degree that EI fits present-day theories of intelligence (i.e., the CHC model), any subsidiary factors of EI reasoning should correlate highly with one another. These and other considerations led to a revision of the original Mayer-Salovey-Caruso Emotional Intelligence Test (MSCEIT) to the MSCEIT 2.

**Methods:**

The MSCEIT 2 was developed and tested across 5 studies: Two preliminary studies concerned, first, the viability of new item sets (Study 1, *N* = 43) and, in Study 2 (*N* = 8), the development of a veridical scoring key for each test item with the assistance of Ph.D. area experts. Next, a pilot study (Study 3, *N* = 523) and a normative study (Study 4, *N* = 3,000) each focused on the test’s item performance and factor structure, including whether a four-domain model continued to fit the data in a manner consistent with a cohesive broad intelligence. Study 5 (*N* = 221) examined the relation between the original and revised tests.

**Results:**

The studies provide evidence for factor-supported subscale scores, and good reliability at the overall test level, with acceptable reliabilities for 3 of the 4 subscale scores, and adequate measurement precision across the range of most test-takers’ abilities.

**Discussion:**

Overall, the MSCEIT 2 used updated theory to guide its construction and development. Its test scores fit the CHC model, and correlate with the original MSCEIT. The revised test is 33% shorter than the original.

## 1 Introduction

Emotional intelligence (EI) concerns how well people reason about emotions and emotional information (Salovey and Mayer, 1990; Mayer and Salovey, 1997). From this perspective, emotions do not disrupt intellect as many believed (Young, 1936; Shaffer et al., 1940; Locke, 2005), but rather serve as signal systems that convey important information: for example, fear signals threat, happiness signals wishes fulfilled. Each emotion conveys a meaning (Plutchik, 2001). Moreover, some people are better at recognizing and reasoning with emotions than others. Although the idea of EI as a potential intelligence was initially greeted with skepticism (e.g., Davies et al., 1998), the skepticism softened as ability-based tests were trialed and evidence for their validity accrued (e.g., Zeidner et al., 2001). We note that a second use of the emotional intelligence term exists, referring to a constellation of socioemotional attributes such as optimism and motivation (e.g., Ashkanasy and Daus, 2005; Cherniss, 2010; Mayer and Caruso, 2025; Schlegel et al., 2013), but the ability approach is our focus here.

### 1.1 Theoretical developments in the study of EI

We view the theory of emotional intelligence as an explanatory tool that can be used to understand the problem-solving abilities people draw on when making sense of the often emotional environment around them. Over the past 35 years, discussion of the theory and empirical findings both argued for revision of some parts of the original theory. We describe our current theoretical outlook on EI and its influence on the transition from the MSCEIT to a revised version, the MSCEIT 2. Note that a 2003 paper had referred to the original MSCEIT as the “MSCEIT V2.0” (Mayer et al., 2003) but the “V2.0” was subsequently dropped. When we use the MSCEIT 2 name here it refers exclusively to the current 2025 revision.

#### 1.1.1 A revised theoretical perspective on emotional intelligence as an intelligence

The 1990 EI theoretical statement acknowledged that the skills we described might not constitute a “coherent construct” (Salovey and Mayer, 1990, p. 201). As empirical tests proceeded, however, the idea showed increasing promise (Mayer et al., 1990; Mayer and Geher, 1996) and by the end of the 1990s EI could plausibly be viewed as a coherent, cohesive group of abilities (Mayer et al., 1999). Over the same period, a new model of human intelligence—the Cattell-Horn-Carroll model (CHC)—was introduced that has guided much of the field of intelligence since. The CHC model views intelligence as a hierarchy with general intelligence at the top and broad intelligences such as visuo-spatial, mathematical, and verbal-propositional intelligences nested below it (Carroll, 1993; McGrew, 2009). Empirically, EI turned out to be one of those broad intelligences (MacCann et al., 2014; Bryan and Mayer, 2020, 2021; Evans et al., 2020).

The fact that EI fits within the CHC model helps contextualize emotional intelligence within the broader study of intelligence(s) (e.g., Schneider et al., 2016; Bryan and Mayer, 2021). In many respects its fit raises the profile and importance of the ability. It suggests—as earlier findings have borne out—that EI will correlate in the *r* = 0.60 range with other broad intelligences (once adjusted for unreliability within a factor model). It also suggests that EI will exhibit incremental validity over other broad intelligences in predicting key outcomes, such as academic achievement, job performance, the quality of social relations, and psychological health. This is also the case (Marder et al., 2004; Lopes et al., 2005; Hertel et al., 2009; Joseph et al., 2015; MacCann et al., 2020). It further entails that any subsidiary factors of EI will be highly correlated and clearly belong to the construct—a matter we assess in factor analyses of the MSCEIT 2 here.

#### 1.1.2 A revised perspective regarding item scoring

Related to measurement, a second change in our perspective concerns the way test responses are scored for correctness. We initially argued, given that emotional expressions and language had evolved (Ekman, 1993; Darwin, 1872/2024), that emotional intelligence items could be scored for correctness according to a general consensus (Mayer et al., 2001; Mayer et al., 2002). Others argued, however, that a general consensus might fail to identify test-takers who were particularly adept at understanding emotions. For the original MSCEIT we therefore recommended a scoring system that employed a consensus among experts, each of whom read through the test and individually identified what they regarded as the correct answers (Mayer et al., 2002). For example, if 81% of the experts chose an alternative as correct and so had the test-taker, the test-taker would receive a score of 0.81 on the item (Mayer et al., 2003).

Researchers remained concerned about scoring, and three further approaches were advanced. A discarded approach was “target scoring” for which test-takers were asked to gauge a target person’s emotional responses to situations. But only some questions about emotions can be scored this way, and some target individuals may respond idiosyncratically, reducing the utility of the scoring system (Mayer and Geher, 1996; Mayer et al., 1999). A further alternative was keying answers to a specific theory. MacCann and Roberts (2008), for example, keyed the correct answers of their Situational Test of Emotional Understanding to a specific theory of emotion appraisal. The issues with using a single theory, however, include that not everyone is likely to agree that the theory is optimal, and psychological theories often exhibit good but imperfect fit to empirical findings—leading to potential mis-scorings. Roberts et al. (2001), however, had suggested yet another approach more similar to that used by established intelligence tests, which they referred to as “veridical scoring.” Our interpretation of veridical scoring involved developing items keyed to emotions research, and then convening experts who would (a) have access to a corpus of key emotional research studies to consult, (b) be able to discuss their answers with one another, allowing them to (c) consider differences of interpretation of test items that test-takers might have; and (d) listen to different opinions before finalizing their own judgments as a group. In a further departure from the expert consensus approach (e) in cases where experts did not agree, the problematic items were removed from the test.

#### 1.1.3 A revised perspective on the content of the four EI domains

The original four-domain model divided EI into four abilities: (a) to perceive, appraise, and express emotions accurately, (b) to access and generate feelings when they facilitate thought, (c) to understand emotion and emotional knowledge, and (d) to regulate emotions to promote the well-being of oneself and others (Mayer and Salovey, 1997). Although we retained the four original domains here, we broadened the inclusion of the skills within each area (e.g., Mayer et al., 2016). This expanded view argued for any new test to include a larger number of briefer item sets than before, recognizing that not all abilities can be assessed in one instrument, but rather, the full spectrum of emotional reasoning can only be sampled. We also renamed “Facilitating Thought” to “Connecting Emotions”—the ability to relate emotion features to similar features of bodily sensations, visual stimuli, and other modalities (Mayer et al., 2024).

#### 1.1.4 A revised perspective on the structure of the test

Any meaningful change to a test entails the possibility that the test structure—that is, the subsidiary factors it measured—might change. Moreover, our aforementioned view of EI as part of the CHC model entailed that the subfactors of EI should plainly belong to EI and correlate sufficiently highly with one another (e.g., *r* from 0.60 upwards), as to be part of the same broad intelligence (see Bryan and Mayer, 2020). In other studies, we reanalyzed four EI tests at the item level and discovered that a three- or four-factor model might not suffice to characterize the earlier tests (Mayer et al., 2024).

Part of the issue may have been due to what Legree et al. (2014) identified as a confounding artifact of the original MSCEIT: The MSCEIT used Likert scale responses across three of the four original domains (e.g., “1 No happiness…3 Some happiness…5 High happiness”). But test takers regularly exhibit individual differences in Likert scale use, with some prone to use higher or lower parts of the response continuum, apart from true differences in judgments, generating nuisance factors. Only the Understanding domain used a multiple-choice format. To reduce artifactual factors, the MSCEIT 2, by comparison, relies chiefly on multiple choice and rank-order responding. In the few remaining instances for which Likert-type responses were regarded as best, they were formatted as multiple choice.

We recognize that investigators also have analyzed the original MSCEIT at the level of its eight tasks; some researchers have concluded that the test consists of three factors (e.g., Palmer et al., 2005; Fan et al., 2010). Acknowledging that possibility, analyses at the item or item parcel level (at which level the original MSCEIT analyses were carried out) might be more informative than task scores, which may cover up the behavior of subsets of items. We use an item-level approach here.

#### 1.1.5 A revised perspective on test reliability

We initially were focused on the original MSCEIT’s reliability for test-takers as a group. But Zeidner et al. (2001, p. 268) pointed out that the test measured test-takers with lower levels of EI with relative precision but was comparatively imprecise for participants with higher ability. Consequently, we also monitored the MSCEIT 2’s precision of measurement across the ability spectrum as the instrument was developed.

#### 1.1.6 A revised perspective on test length

Finally, the time pressures on assessment professionals, coupled with people’s shorter attention spans due to the influence of technologies such as smart phones and online content have created a demand for shorter measures (Mendoza et al., 2018; Firth et al., 2019; Chu et al., 2021). In response, test developers have worked to reduce test lengths. A concern with such brevity is that the reliabilities of subscales can trend lower than is desirable (Kruyen et al., 2013; Ziegler et al., 2014), but the tradeoff is that shorter tests often exhibit equivalent or higher relations with external criteria (e.g., Burisch, 1984; Credé et al., 2012; de Vries, 2013; Ziegler et al., 2014). At 141 items, the original MSCEIT was relatively lengthy and required extended concentration; we sought to reduce its length.

### 1.2 Rationale for the revision, goals, and research plan

The rationale for developing the MSCEIT 2 was to create a test that reflected the theoretical advances just described. That is, our goals were to create a test that (a) measured EI as a broad intelligence that (b) included a more diverse set of emotional problems to solve, and (c) employed veridical scoring to determine correct answers. Further, we (d) employed more uniform response scales across domains to clarify the test’s factor structure, (e) used IRT to clarify its precision across ability levels, and (f) created a shorter test. Given the scope of such changes, we anticipated that the MSCEIT 2 would better represent the construct and that its overall EI score would be somewhat distinguishable from the original MSCEIT score, correlating in the *r* = 0.60 to 0.90 range. Although not specifically a part of the theoretical changes, care was taken to ensure test fairness throughout the development process: from item construction, to recruiting diverse test-takers, to checks to ensure item performance was equitable across the groups tested.

**Figure 1:**
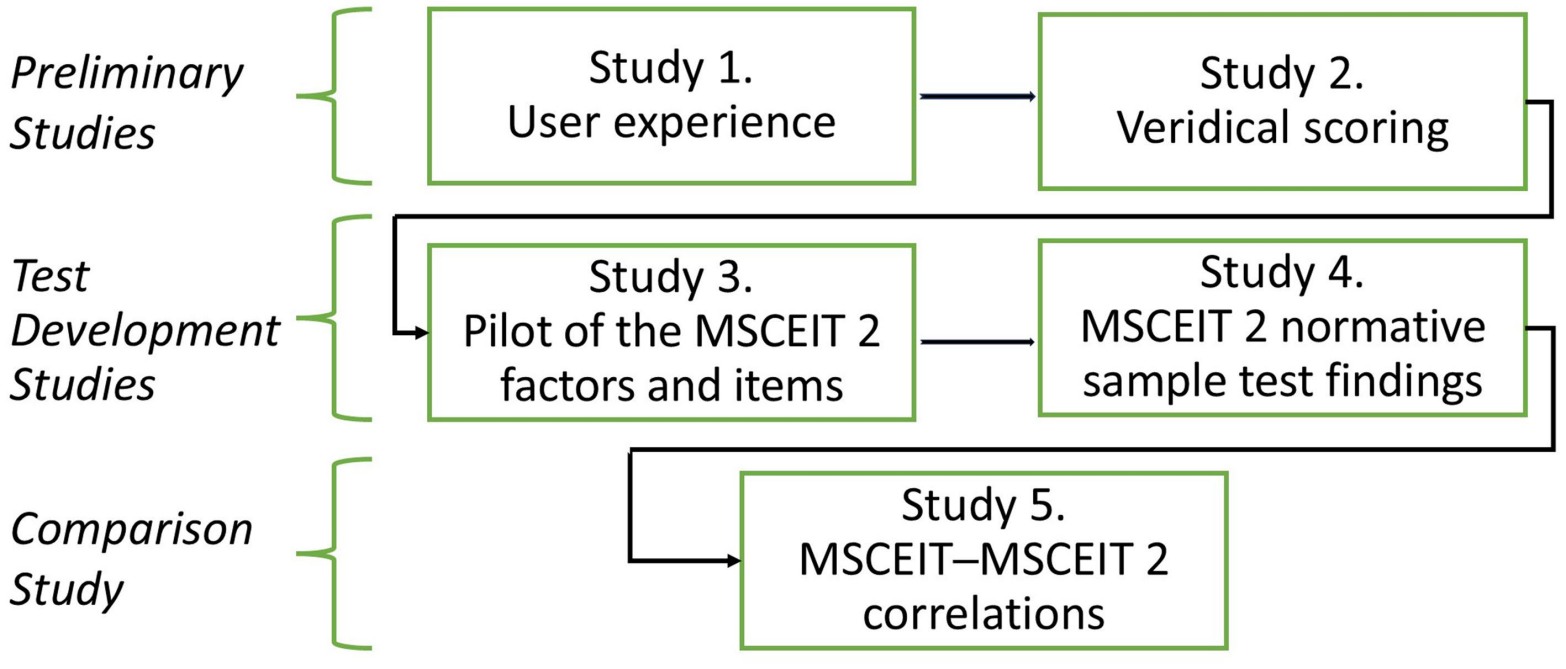
An overview of the research here: two preliminary studies, two main studies of the MSCEIT 2 item sets, and a study of the MSCEIT and MSCEIT 2 test score relations.

The research presented here involves five studies indicated in Figure 1, including two preliminary studies, two studies of the test’s item sets, employing pilot and normative samples, and a comparison of the original MSCEIT and MSCEIT 2.

## 2 Development of the MSCEIT 2

### 2.1 Test content and response processes

The validity evidence for a test begins with its content and the kind of mental processes the questions elicit (e.g., Loevinger, 1947; Cronbach and Meehl, 1955; Messick, 1995; Joint Committee, 2014). Test items for the MSCEIT 2 were developed to match the four-domain model of emotional intelligence, as had been done for the MSCEIT—but, as noted, the variety of items that might reflect such abilities had grown since the original four-domain model. For that reason, the number of item sets were expanded from 8 tasks to 12 question types. As before, all items were designed to elicit mental problem-solving to ensure evidence for validity from response processes (e.g., Messick, 1995; Joint Committee, 2014; Hubley and Zumbo, 2017; Mayer and Bryan, 2024). Item development began with interviews of MSCEIT test administrators and test-takers to identify areas for improvement from the MSCEIT to the MSCEIT 2. For example, test administrators requested clearer instructions for how test-takers should approach certain tasks. Next, a literature review of each of the four problem-solving domains was conducted; based on that research, new items were written, ensuring there was a mix of genders, ethnic backgrounds and emotions. An expert panel, described in the veridical scoring section below, provided feedback on item wording, use of jargon, and of colloquial expressions, and also provided feedback on possible issues of bias relating to culture, gender, and race/ethnicity.

An overview of the MSCEIT 2 question types for each of the four domains can be found in Table 1. The User Experience Study, the first of the two preliminary studies, is reported next.

**Table 1:** The emotional intelligence question types of the MSCEIT 2.

Question type	Description
Perceiving emotions: The accuracy with which people perceive emotions in visual and other modalities	
Videos	Identify an emotion expressed by a person’s facial expression from a brief video.
Contextual pictures	Identify an emotion a person is experiencing from an outline sketch of the person’s posture.
Faces*	Identify emotion(s) in a photograph of a person’s face appearing after an image of the same person’s neutral expression.
Connecting emotions: The ability to relate features of emotions across modalities such as to visual stimuli and bodily sensations	
Emotion dimensions	Match an emotion to its level of energy and pleasantness described on a graphic scale.
Changing contexts	Infer what task a person should work on next to be effective, given a change in their emotions.
Facilitation*	Given an emotional state, pick the task at which a person will be effective.
Sensations*	Describe an emotion in terms of its physiological sensations.
Understanding emotions: Identifying information conveyed by emotions and emotional signals	
Changes	Identify how a person’s emotion might change after an event.
Blends*	Identify two or more emotions that, when combined, produce a complex emotion.
Progressions*	Order emotions according to the intensity of energy or pleasantness.
Managing emotions: guiding emotions in oneself and others	
Emotion scenarios*	Read a vignette of people interacting and choose the response that indicates an effective way to achieve a targeted emotional outcome.
Picture panels	View a brief visual narrative of people interacting and choose the response that indicates an effective way to achieve a targeted emotional outcome.

*Question types with asterisks appeared on the original MSCEIT in the same domains (but no individual items were carried over from the original version). Sensations questions only included physiological sensations rather than color, light, temperature included in the MSCEIT. In addition, the MSCEIT Perceiving area contained Pictures questions, consisting of nature scenes and abstract designs. The MSCEIT Managing areas of Managing Emotions (in the self) and Emotion Relations were combined (with all new items) to make Emotion Scenarios for the MSCEIT 2.

## 3 Preliminary empirical studies

### 3.1 Study 1: the user experience study

**Figure 2:**
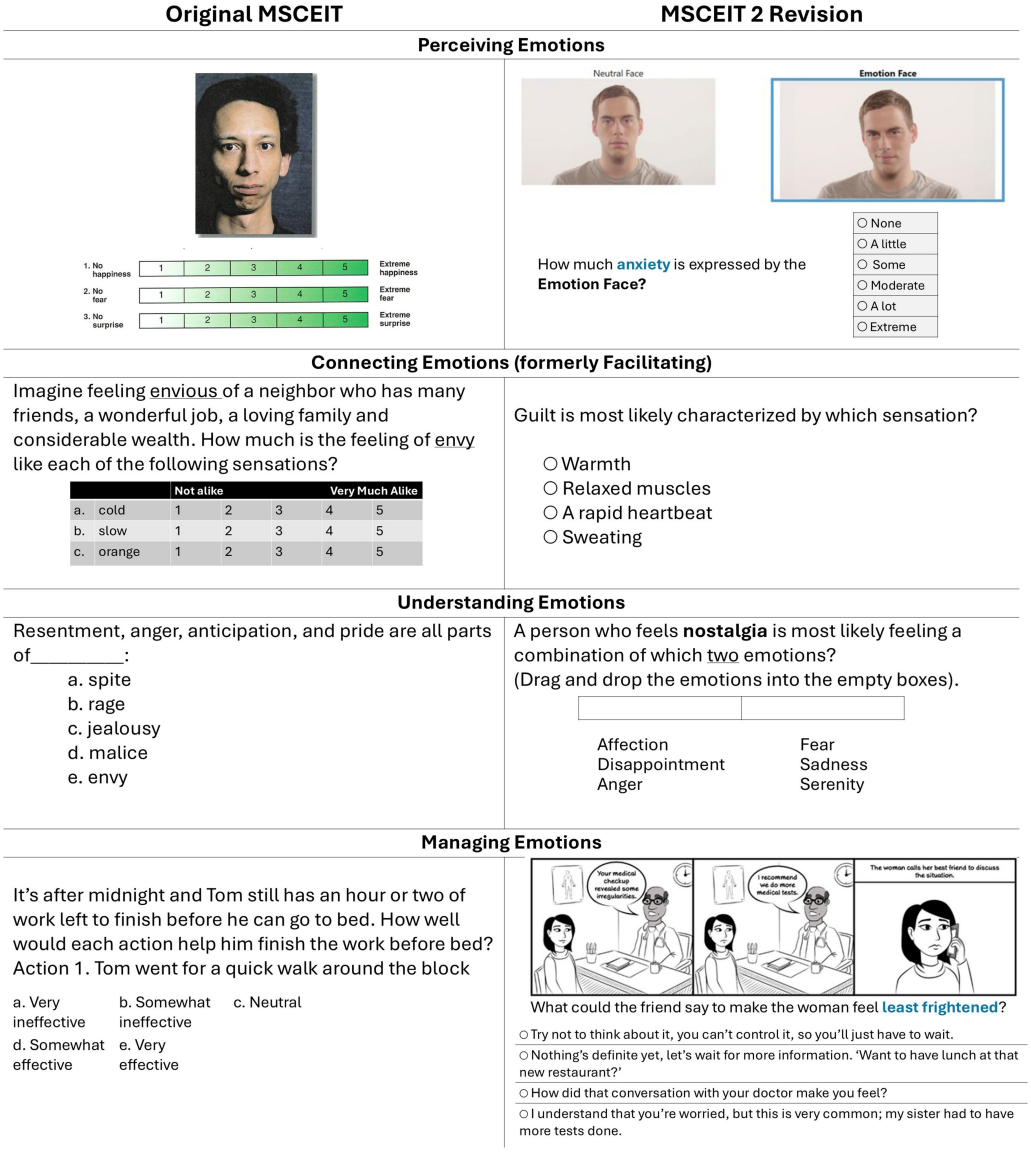
Representative items from early versions of the MSCEIT and MSCEIT 2, respectively. The MSCEIT 2 revision attempts to improve test-taker engagement and to employ uniform response scales. Some item formats have been adjusted in the figure for greater readability.

To ensure the 12 question types were clearly understood by test takers, a User Experience Study presented the new question designs from a preliminary set of MSCEIT 2 items to 43 adults (18 years or older) drawn from a general population of varied age and gender (Lin and Pothera, 2019). They completed the test while their behavior was observed and then engaged in a post-administration interview. Participants indicated whether the online implementation of the test was well designed, clear, matched the skills they used in their daily lives, was of an appropriate difficulty level, and indicated any other concerns. Researchers recorded their responses and timed the test session. Revisions were implemented to improve the clarity of the instructions and simplify questions that test-takers remarked were “overwhelming.” The sizing of images and videos and other online conventions were improved as well. Guided by this feedback, new test items were generated. Figure 2 contains examples of item types on the MSCEIT and their revised forms on the MSCEIT 2.

### 3.2 Study 2: the veridical scoring approach

Once the initial set of 159 MSCEIT 2 items was finalized, an expert panel was convened to implement veridical scoring (see Section 1.1.2). The panel members were eight Ph.D. psychologists with a track record of published research in the area and academic appointments in institutions in North America (*n* = 2), Asia (1), Europe (4), and Australia (1), including four women and four men. The experts were given 12 to 22-page scoring manuals for each domain, assembled based on a review of relevant literature. To begin the veridical-scoring process, the experts used their best judgment, with reference to the manuals, to indicate the correctness of each response option to the initial MSCEIT 2’s 159 items: either a 2 (full credit), 0 (no credit), or, for some items, 1 (partial credit). Their evaluations were compiled and where there were disagreements, responses were discussed among the group, including at an in-person meeting of four members. After the MSCEIT 2 Pilot Study data collection (Study 3 here), several further items were flagged as problematic, and the committee discarded six items. Panel members received an honorarium, and four members who met in-person at MHS (the test publisher’s offices in Toronto, Ontario) during the final stages of item scoring had their travel expenses paid for. The result was a new system for which each item was carefully considered and discussed by the expert panel, and items that failed to possess clear answers were discarded.

## 4 Overview of the main MSCEIT 2 studies

After the MSCEIT 2 initial item set and scoring were established, the Study 3 pilot data and Study 4 normative sample data were collected. As noted in sections 1.1.1 and 1.1.4, the test’s factor structure and whether a four-domain model fit it would speak both to the compatibility of the MSCEIT 2 with the CHC model of intelligence, and to the validity of the four-domain structure and scores. We therefore tested factor models in both the pilot and normative data samples. Study 5 reports the relation between the MSCEIT 2 and the MSCEIT.

## 5 Study 3: Pilot test of the MSCEIT 2 factors and items

Study 3 assessed the overall functioning of the initial 153 MSCEIT 2 items (159 minus 6 discarded by the expert panel) and explored the factors the items measured. We expected that: (a) there would be an overall EI factor represented by most items loading positively on a single factor, and that (b) after selecting the items that loaded positively on that overall factor, the data would fit a 4-factor confirmatory model using the items’ *a priori* assignment to a given domain.

### 5.1 Method

#### 5.1.1 Participants

Participants were 523 test takers representative of US population demographics composed of 274 women and 248 men (and 1 non-respondent), with between 91 and 130 participants each across age groups of 20s, 30s, 40s, 50s, and 60-plus. The participants self-identified as 8.0% Asian, 10.0% Black, 10.7% Hispanic, 68.1% White, and 3.1% in other groups or not specified, with educational levels at 29.6% high school or below, 54.3% with some college or college degree, and 16.1% with postgraduate degrees (see the Technical Supplement for details; Mayer et al., 2024).

#### 5.1.2 Procedure and participant screening

The MSCEIT 2 Pilot Study (Study 3) survey was constructed on *Survey Gizmo* (now *Alchemer*) and administered using *Prime Panels* (e.g., Chandler et al., 2019). Question types were presented in two counterbalanced orders, as two separate surveys. Of 3,032 logons to the surveys, 2,068 completed the test. Participant recruitment continued until all demographic groups, including cross-tabular criteria (e.g., age x ethnic group) were filled according to a stratified sampling plan. A randomized selection procedure was employed to reduce over-sampled categories of participants (e.g., McNemar, 1940; McCready, 1996; Mweshi and Sakyi, 2020), leading to a final representative sample of *N* = 523 on which all further Pilot Study analyses were conducted.

### 5.2 Results

#### 5.2.1 Preliminary analyses

##### 5.2.1.1 Review of item content

Post-data-collection, the research team carried out a further qualitative review of the test items and flagged eight items that appeared to be mis-scored, unclear, or otherwise poorly worded; these were referred to the expert panel, who recommended removal of six of the eight flagged items, leaving 153 items (see Section 3.2).

##### 5.2.1.2 Initial factor analysis of the MSCEIT 2 structure

All factor analyses reported here were conducted in Mplus (Muthén and Muthén, 2017). The data were treated as categorical and factors were estimated using a weighted least squares mean and variance adjusted method (WLSMV).

##### 5.2.1.3 Checking for a first factor

We conducted a first EFA of the 153-item set to determine the degree to which items reflected a general factor of emotional intelligence. Of the 153 items, 137 items loaded above zero suggesting a general factor. See Table 2 for this and other model fits.

**Table 2:** Exploratory and confirmatory simple structure factor analyses of the MSCEIT 2 pilot study data (*N* = 523)^a^.

Model	Items	Free params	χ^2^	df	RMSEA	CFI	TLI	SRMR
*K* = 153 Item exploratory factor analyses^b^								
1-Factor EFA	153	153	12633.77	11,475	0.014	0.897	0.896	0.085
2-Factor EFA	153	305	12084.40	11,323	0.011	0.932	0.931	0.079
3-Factor EFA	153	456	11769.52	11,172	0.010	0.947	0.945	0.075
4-Factor EFA	153	606	11542.07	11,022	0.009	0.954	0.951	0.073
*K* = 111 Item confirmatory factor analyses								
1-Factor, Global EI	111	260	6928.70	5,994	0.017	0.924	0.923	0.079
2-Factor visual v. verbal	111	261	6876.35	5,993	0.017	0.928	0.927	0.078
3-Factor (omitting Conn.)	89	211	4450.95	3,824	0.018	0.942	0.940	0.078
4-Factor, four domains	111	266	6652.65	5,988	0.015	0.946	0.945	0.076

a The exploratory factor analysis was conducted with the K = 153 item set; the confirmatory factor analyses, constrained to simple structure, on the 111 items. Both the EFA and CFA defined the data as ordered categorical, and used weighted least squares mean and variance adjusted (WLSMV) estimator in Mplus.

b The item-level exploratory models, with the exception of the 1-Factor model, yielded solutions that were not always clear and showed only hints of the confirmatory models successfully fit here.

##### 5.2.1.4 Problematic question types

We expected that most question types—sets of similar items described in Table 1—should, when analyzed separately, be predominantly unifactorial, defined as 75% or more of their items loading positively on the set’s first factor (of two or three factors). Eight of 12 question sets fit that criterion, but Faces, Videos, Changing Contexts, and Facilitation did not.

##### 5.2.1.5 Modifications to scoring

Faces items appeared to split according to whether the items concerned an emotion that was present or absent in the picture. The decision that a specific emotion is absent draws on a different response style than does the decision that it is present, according to earlier factor analyses (Føllesdal and Hagtvet, 2009; Mayer et al., 2024). Because good emotional perception involves accuracy in both, to adjust for differences in response tendencies we centered each participant’s use of the multiple-choice options (which, for example, could include “no contempt” to “extreme contempt”), by adjusting their score according to the respondent’s customary use of the scale (e.g., whether they inclined toward “extreme” emotion or to “no” emotion responses) as indicated by their median response. After this rescoring, Faces appeared closer to unifactorial than before. Details are in the Technical Supplement (Mayer et al., 2024, Section 2.1).

On the Changing Contexts questions, by comparison, some participants exhibited a positivity bias such that no matter what question they encountered, they favored a pleasant mood for it. This response style was interpreted as reflecting a reliance on positive thinking, as opposed to method variance, and therefore neither items nor scoring were revised. The task also may have been overly complex (as test-takers noted in the earlier User Experience study), as it exhibited just one item with a positive loading *λ* (lambda) > 0.40 on both the original EFA and later confirmatory factor analysis (CFA).

On Videos, the less-than-ideal fit to a single factor appeared due to the items being too easy, which reduced their discrimination. The veridical panel had given some responses partial credit (e.g., 0 = no credit, 1 = partial credit and 2 = full credit). Items were made more difficult post-hoc by reassigning partial credits as no credit.

##### 5.2.1.6 Test of a 1-factor model

We then conducted a confirmatory factor analysis to check whether a 1-factor model might represent an overall emotional intelligence. The model appeared almost good enough to accept as it was, with an RMSEA of 0.014, and CFI and TLI of 0.897 and 0.896, suggesting the near-unitary nature of the area (see Table 2, top rows). We note, however, that our *N* = 523 led to a participants-to-indicator variable ratio of approximately 3:1, which fell short of the 5:1 recommended by many experts, although other issues are relevant as well (e.g., Flora and Flake, 2017; Gorsuch, 2015; Nunnally, 1967, p. 436).

#### 5.2.2 Tests of the a priori confirmatory models on the pilot *K* = 111 item set

A further goal was to reduce the test length from the *K =* 137 items that loaded on the general factor above zero (see section 5.2.1.3). To do so we began by removing a number of poorly-functioning items, operationalized as items loading *λ* < 0.20 on the first overall factor of the test, using the 1-factor EFA, resulting in an item pool of *K* = 111. Five items of those 111 were replaced with items loading lower than λ = 0.20 to supplement tasks such as Videos that were engaging for test-takers but that had low item counts, as well as to balance “emotion present” versus “absent” items on Faces.

EI research established several preferred factor models for the mental ability—*a priori* 1-, 2-, 3- and 4-factor models (e.g., Mayer et al., 2002; Palmer et al., 2005; Legree et al., 2014; MacCann et al., 2014). Briefly, a 1-factor model tests the idea of a unitary EI by fitting all the items to a single factor. The 2-factor model we employed here compared visual (e.g., Videos) with verbal items (e.g., Emotion Scenarios). The 3-factor model employed all the item sets a priori-assigned to the Perceiving, Understanding, and Managing domains (see Table 1), excluding those reflecting the Connecting Emotions domain. The 4-factor model corresponded to the four-domain model of EI (including Connecting Emotions). Detailed diagrams of the models tested can be found in the Technical Supplement (Mayer et al., 2024, Section 1.3).

We fit all four models to the 111 items in a series of confirmatory analyses. Each test was conducted using a simple-structure model in which each item was constrained to load only on its assigned ability factor. The four models all fit the data reasonably well, and the fit improved slightly from the 1-factor to the 4-factor model (Table 2, lower rows). Associated research on CHC broad intelligences (e.g., verbal, visuo-spatial, etc.) indicates that their estimated correlations (e.g., between verbal and spatial intelligences) averages about 0.60 (Bryan and Mayer, 2020). For that reason, subsidiary factors within a broad intelligence should range from *r* = 0.60 upwards. For the best-fitting 4-factor model, the estimated correlations among the domains ranged from *r* = 0.66 (Perceiving with Managing), to *r* = 0.90 (Understanding with Managing) which fell within the targeted range.

#### 5.2.3 Descriptive statistics

Descriptive statistics for participants’ overall and domain scores are indicated at the top of Table 3. The 111-item form yielded a range of scores from 45 to 200 with the mean score of 138.87. The mean proportion correct score, relative to the maximum obtained score, was 0.69. The scale exhibited a slight negative skew of −0.70, with scores more concentrated at the higher end of the distribution.

**Table 3:** Selected descriptive statistics of the MSCEIT 2 pilot and normative sample scores.

	Items	Obtained min-max	Mean points^a^	Proportional score^b^	Std. dev.	Skew	Alpha reliability
Study 3. Pilot study *K* = 111 items *N* = 523^a^							
MSCEIT 2 overall	111	45 to 200	138.87	0.69	30.88	−0.70	0.93
Perception	36	17 to 78	49.92	0.63	11.11	−0.56	0.77
Connect	22	4 to 33	20.92	0.63	5.81	−0.48	0.68
Understand	31	4 to 58	38.45	0.66	11.04	−0.85	0.84
Manage	22	7 to 43	29.59	0.70	8.22	−0.76	0.84
Study 4. Normative sample study *K* = 107 items *N* = 3,000^a^							
MSCEIT 2 overall	107	38 to 183	126.05	0.69	26.42	−0.62	0.90
Perception	35	5 to 70	43.76	0.63	10.36	−1.12	0.77
Connect	20	5 to 37	21.61	0.58	21.61	−0.31	0.53
Understand	30	4 to 55	31.80	0.58	31.80	−0.34	0.80
Manage	22	6 to 43	28.88	0.67	29.88	−0.69	0.79
Study 4. Normative sample study *K* = 83 items *N* = 3,000^a^							
MSCEIT 2 overall	83	32 to 143	95.85	0.67	21.13	−0.59	0.88
Perception	24	2 to 48	29.22	0.61	7.60	−1.07	0.70
Connect	17	2 to 32	18.41	0.58	4.95	−0.31	0.56
Understand	24	2 to 44	24.49	0.56	8.12	−0.27	0.75
Manage	18	3 to 35	23.73	0.68	6.09	−0.77	0.75

a Descriptive statistics were calculated for the scored summed points of a given scale. Item scores in the Pilot Study (Study 3) were recoded downward no credit (0), partial credit (1), and full credit (2) to match the Normative Sample Study, from original scores values of (1), (2), and (3), to allow for more convenient comparisons between study analyses.

b To further facilitate comparisons, the Proportional Score indicates the Mean Summed Points of the scale divided by the maximum obtained score.

We next created scales based on an earlier, *a priori* assignment of item sets we had made to the four domains and calculated their reliabilities for both the *K* = 153 and 111 versions of the test. Because model fits supported the general integrity of a 4-factor approach, coefficient alphas were appropriate for testing the factor-based scale reliabilities. For the *K* = 153 and 111 item sets respectively (only the *K* = 111 alphas are in Table 3), the coefficients were *α* = 0.91 and 0.93 for the whole test, *α* = 0.75 and 0.77 for Perceiving, 0.54 and 0.68 for Connecting, 0.84 and 0.84 for Understanding, and 0.84 and 0.84 for Managing. These findings are considered further in the Discussion.

### 5.3 Discussion of the pilot study findings

The first phase of our analyses of the MSCEIT 2 involved an examination of the question sets in terms of the adequacy of their scoring. Specifically, the Faces items split into two factors depending upon whether items asked about emotions in the face that were present or absent. This divide is a common issue in the MSCEIT series of tests for Face perception (e.g., Mayer et al., 2014; Mayer et al., 2024). We therefore rescored the Faces items to remove that scale usage issue as much as possible (for details, see Mayer et al., 2024). The Videos items appeared too easy, so we made Videos more challenging by removing partial-credit items. After trimming all the items to remove those that loaded *λ* < 0.20 on the first unrotated factor of the *K* = 153 test (rescored as above), we arrived at a 111-item set.

A test of confirmatory *a priori* factor models indicated that all models fit reasonably well, albeit the 4-ability theoretical model fit best. One drawback of the 4-factor model was the estimated correlation of *r* = 0.90 between the Understanding and Managing domains. The reliabilities of the *a priori* scales were generally good in both the *K* = 153 and 111 item sets, but the Connecting Emotions area fell short of conventional standards for reliability, with the α falling at 0.54 for the *K* = 153 and 0.68 at the *K* = 111 item sets. The domain was retained, however, because evidence indicated that the corresponding factor likely existed (Mayer et al., 2024) and was therefore better to measure than not. These 111 items formed the starting version of the MSCEIT 2 administered to the normative sample.

## 6 Study 4: MSCEIT 2 normative sample test structure, reliability, and test information curve

### 6.1 Overview and preregistered hypotheses

In Study 4 we analyzed data from a new group of participants who took the *K* = 111 MSCEIT 2 test items, with the continued purpose of understanding the factor structure of the MSCEIT 2 and the nature of its scales. This information, in turn, informs both its viability as a broad intelligence, and the potential to create factor-valid subscales. In the process, we hoped to finalize an item set, create a measure with meaningful subscales, ensure its fairness, and shorten its length.

Five hypotheses were preregistered on the Open Science Foundation website at https://osf.io/nmp68/, of which the 1st, 2nd, and 5th were tested here. Hypothesis 1 was that the MSCEIT 2 could be described by a 1-factor simple-structure model with fit statistics of RMSEA < 0.06 and CFI and TLI > 0.92. The slightly relaxed criteria for the CFI and TLI values reflected the higher statistical noise expected when analyzing test items as opposed to scales, owing to the larger number of items (e.g., Little et al., 2002, 2013). Hypothesis 2 stated that we would successfully fit 2-, 3-, and 4- *a priori* factor models to the data, with the caveat that we might encounter fairly high correlations among domains and possibly also Heywood cases (estimated correlations > 1.0). Hypothesis 5, tested on the final form of the MSCEIT 2, was that self-estimated EI scores would correlate *r* ≤ 0.25 with MSCEIT 2 scores. An additional two hypotheses of the preregistration (3 and 4 in the original document) were not tested here. One was a backup needed only if Hypotheses 1 and 2 both failed; the other concerned ancillary rational scales that are not examined here for reasons of length.

### 6.2 Materials and method

#### 6.2.1 Sample characteristics

The *N* = 3,000 normative sample included both Canadian (*n* = 300) and U.S. constituents (*n* = 2,700) approximately reflecting the ratio of the two nations’ populations. The overall sample contained equal numbers of women and men and was drawn 20% each from five age ranges: 18 to 29 years, 30s, 40s, 50s, and 60 years or older. The U.S. sample was divided among those who identified as Asian (6%), Black (12%), Hispanic (16%), White (63%) and Other (3%); the Canadian sample identified as 21% Visible minority and 79% Not visible minority (the racial/ethnic group categories used by the Canadian census). The U.S. and Canadian samples, respectively, reflected a range of educational attainment levels that mirrored similar proportions in their national populations: “High School or less” (37 and 35%), “Some college” (30 and 35%), “Bachelors” (21 and 21%), and “Graduate Degree” (12 and 9%). Both samples were approximately representative of their respective nation’s geographical regions, matched within 5% of U.S. 2022 and Canadian 2021 census data (Statistics Canada, 2023; U.S. Census Bureau, 2023, Table S0201).

#### 6.2.2 Sample screening

An initial 15,046 individuals had logged onto the survey of whom 6,180 were eligible to participate and completed most of the survey (see also Mayer et al., 2024 for details). As before, the MSCEIT 2 survey was constructed on *Survey Gizmo* (now *Alchemer*) and administered using *Prime Panels* (e.g., Chandler et al., 2019). Participant recruitment continued until all demographic groups, including cross-tabular criteria (e.g., gender x education) were complete. From those 6,180 participants, 867 cases were removed for signs of poor compliance, including (a) completing the survey in under 12 min, (b) failing one or more attention checks, (c) leaving 10% or more of the questions unanswered, or (d) repeating a single response choice on the MSCEIT 2 more than 50% of the time. After cleaning the data, 5,313 participants with valid protocols remained. The final *N* = 3,000 were randomly selected from the group to match census quota targets.

#### 6.2.3 Self-estimated emotional intelligence

A brief self-estimated EI scale designed to assess the 4-domain areas of reasoning was also included after the MSCEIT 2 items. The scale consisted of 7 items for each of the four ability domains (28 items total). Examples included “I am an expert at reading other people’s emotions” (Perceiving Emotions) and “I manage my emotions well” (Managing Emotions), with all phrased in the direction of higher answers reflecting more EI, and answerable on a Likert-type scale from 1 “Not at all like me” to 5 “Describes me very well.”

### 6.3 Results

#### 6.3.1 Descriptive statistics

Descriptive statistics for participants’ overall and domain scores are indicated at the top of Table 3. Beginning with the 111 items, we removed 4 items because they had been miscoded in the Normative dataset (see Mayer et al., 2024, Section 4.1, for details). The 107-item form yielded a range of scores from 38 to 183 (with a possible range of 0 to 214), with the mean score of 126.05 and a mean proportional score of 0.69 of the maximum obtained score, and an alpha reliability of *r* = 0.90. The scale exhibited a slight negative skew of −0.62, with scores more concentrated at the higher end of the distribution. We next tested Hypotheses 1 and 2 concerning the factor structure of the MSCEIT 2. Because the EI models we test here are regularly tested and confirmed on EI scales both by our own and other research groups (e.g., Mayer et al., 2002; Fan et al., 2010; MacCann et al., 2014), we modeled the full sample rather than employing a hold-out sample.

#### 6.3.2 Did a 1-factor model fit? (Hypothesis 1)

Our first hypothesis was that a 1-factor model would fit the normative sample at the item level. The fits of the factor model to the *K* = 107 remaining items can be found in the top portion of Table 4. The fit of the general 1-factor EI model was surprisingly poor, and so Hypothesis 1 was rejected.

**Table 4:** Fits for confirmatory factor analyses of the *K* = 107 and 83-item versions of the MSCEIT 2 normative data (N = 3000).

Models	No. of factors	Items	Free params	χ^2^	df	RMSEA	CFI	TLI	SRMR
Simple structure models of the MSCEIT 2 at *K* = 107 items									
General EI	1	107^a^	249	14972.72	5,564	0.024	0.869	0.867	0.055
Visual – Verbal	2	107	250	14475.40	5,563	0.023	0.876	0.874	0.054
No connecting	3	87	206	8097.03	3,651	0.020	0.929	0.927	0.048
4-Domain	4	107	255	10310.60	5,558	0.017	0.934	0.933	0.045
Simple structure models of the MSCEIT 2 at *K* = 83 items									
General EI	1	83	193	10075.69	3,320	0.026	0.870	0.867	0.054
Visual – Verbal^b^	2	83	194	8475.03	3,319	0.023	0.901	0.898	0.050
Experiential – Strategic^c^	2	83	194	8802.24	3,319	0.023	0.894	0.892	0.051
3-Domain; excl. connecting	3	66	157	5149.23	2076	0.022	0.928	0.926	0.047
4-Domain	4	83	199	6788.99	3,314	0.019	0.933	0.931	0.044
Hierarchical model of the MSCEIT 2 at *K* = 83 items									
EI→4 Domains→83 items^d^	5	83	194	8436.95	3,319	0.019	0.932	0.930	0.044

b The visual area consists of Videos, Faces, Contextual Pictures and Emotion Dimensions; the remaining question sets were regarded as verbal.

c We have included a fit for an Experiential-Strategic model (domains 1 and 2 versus 3 and 4) although it was not originally part of our preregistered hypotheses, because it appears in large-scale studies in the literature.

d The EI to Understanding and Management domains were set as equal to remove a Heywood case (see text).

#### 6.3.3 Did 2-, 3-, and 4-factor models fit? (Hypothesis 2)

The second hypothesis was that 2-, 3-, and 4-factor CFA models would fit, albeit the factors might be quite highly correlated among themselves. In fact, the 2-factor solution (Visual-Verbal) exhibited a similar fit to the 1-factor solution. The 3- and 4-factor solutions, however, did exceed our fit criteria. The 4-factor model exhibited an RSMEA of 0.017, and CFI and TLI of 0.934 and 0.933, respectively. The 4-factor fit was especially impressive because the set of 107 items were unscreened for functionality or other issues in this new sample. Also as predicted, the estimated correlations among factors in the 4-factor model were sometimes quite high, indicating their cohesive relatedness as with an *r* = 0.93 between the Understanding and Managing domains.

#### 6.3.4 Reliabilities at *K* = 107 for the standardization sample

The Cronbach alpha reliabilities of the *K* = 107 item set are in Table 3 (right). The EI Total was *α* = 0.90, with three subscale scores ranging from *α* = 0.77 to 0.80, and Connecting lower once more at 0.53—similar to the *K* = 111 Pilot Study (Study 3) values.

#### 6.3.5 Item reduction to 83 items for fairness and function

##### 6.3.5.1 Removal for poor psychometrics, length, and item content

Of the 107 items, a group of further items were flagged for removal for one or more of the following reasons: 6 items because IRT analyses indicated that they were too easy, 6 items to reduce the length of longer question sets, 6 more items because they were the last remaining item of an item set referring to a particular stimulus, such as a face or a text scenario, and 1 item because it was uncorrelated with the rest. Two further items were flagged because of their content (one for being overly negative and the other for being the only item asking about a child) (see Mayer et al., 2024, Section 3.2).

##### 6.3.5.2 Removal to promote test fairness

Seven further items were flagged for removal for reasons of test fairness. We first calculated Differential Item Functions (DIF) via IRT for each of the 107 items, comparing the performances of both women and men, and then for four racial/ethnic groups into which most of the standardization sample could be divided (Asian, Black, Hispanic, and White individuals). Twelve items were flagged on that basis and referred to a committee on test fairness, who recommended, based on the item content and statistical findings, that six of the 12 items be removed. We also calculated the magnitude of observed differences in response frequencies for each item using Cliff’s delta, a measure of group differences appropriate to ordinal data (Cliff, 1993; Neuhäuser et al., 2007); on that basis, a single item was removed owing to its elicitation of different group means.

##### 6.3.5.3 The 83-item set

In all, 24 of the 107 items were removed for one or more of the above reasons, leaving a final 83 items. Means, standard deviations, and other statistics are in the lower portions of Table 3.

##### 6.3.5.4 Factor analyses of the 83 final item set

The factor models were then tested one further time on the final 83 items. The fits are reported in the second and third sets of rows of Table 4 and exhibited considerable continuity from the first set of analyses. This was likely because non- and minimally- functional items often have little impact on fit in our experience.

**Figure 3:**
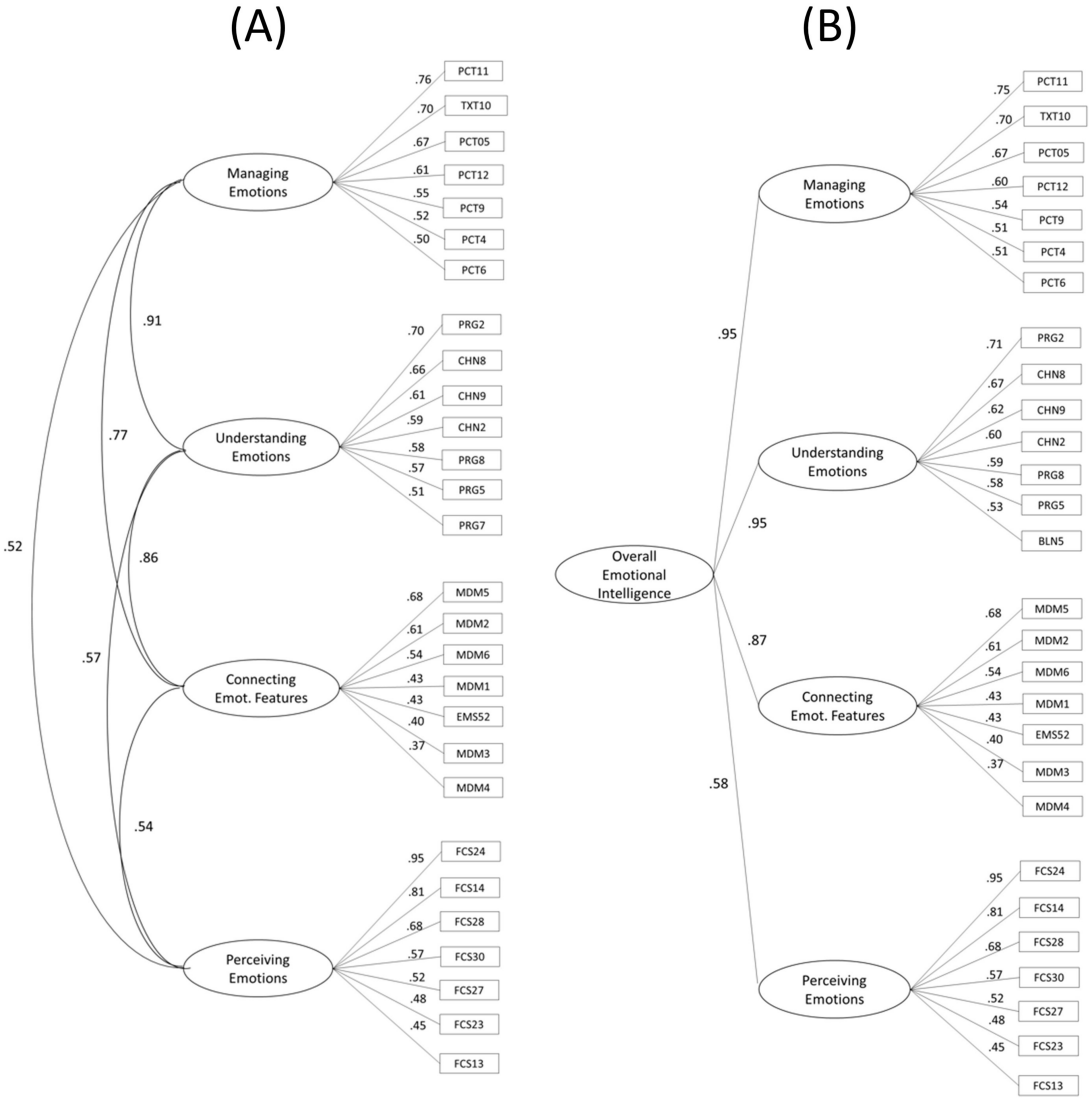
Two variants of the 4-factor model. **(A)** A 4-factor simple structure model of the MSCEIT 2 and **(B)** A hierarchical version of the same model with overall emotional intelligence. Both **(A,B)** show the 7 highest loading items per factor. FCS, Faces; CPX, Contextual Pictures; VDS, Videos; MDM, Emotion Dimensions; EMS, Changing Contexts; SNS, Sensations; PRG, Progressions; CHN, Changes; BLN, Blends; PCT, Picture Panels; TXT, Emotion Scenarios. Portions of the solution in Figures (left) were previously reported in a table as part of joint analysis of four scales of EI (Mayer et al., 2024; Table 5).

As before, the 4-factor model fit best. The model is depicted in Figure 3 (left) and includes the 7 highest-loading items for each domain. It is worth noting that these models employed only the confirmatory *a priori* models; no items were removed even though they violated the model. If we dropped just one item from the 1-factor model, for example, the model would nearly fit. Second, a slightly revised 4-factor model tailored to an EFA of the final dataset might be more viable than the *a priori* version (see Mayer et al., 2024). The goal here, however, was to determine whether a basis existed for the proposed 4-factor model; judging by the model fit, it did. The correlation among the factors ranged from *r* = 0.52 to 0.91.

The Perceiving Emotions domain was dominated by the Faces item set although Videos exhibited low positive loadings not shown here (e.g., *λ* ≤ 0.35). Connecting was dominated by Emotion Dimensions (MDM) and Changing Contexts (EMS) items. Facilitation items loaded positively on the Connecting domain but do not appear because items with *λ* ≤ 0.24 are not shown. Understanding encompassed Changes (CHN), Progressions (PRG), and Blends (BLN), and Management included both Picture Panels (PCT) and Emotion Scenarios (TXT). The solution for all 83 items can be seen in table form in the Technical Supplement (Mayer et al., 2024). Figure 3, right, depicts a hierarchical version of the model with an overall EI added. An initial test of the hierarchical model included a Heywood case of 1.0 between EI and Understanding, which is common in models such as this (Wang et al., 2023). To remove it, we constrained the EI-to-Understanding and EI-to-Management relations to be equal.

#### 6.3.6 Were ability- and self-estimated EI unrelated? (Hypothesis 3; Hypothesis 5 in the preregistered document)

The self-estimated EI scale yielded a reliable overall score of *α* = 0.95, with an average response on the 28 item 5-point Likert scale of *M* = 3.57, *S* = 0.65. The scale correlated *r* = −0.07 with the MSCEIT 2, which was, as hypothesized, below 0.25, although lower than anticipated. More details are in the Technical Supplement.

#### 6.3.7 Reliability revisited

Because the overall EI score is near-unifactorial and the subscales are unifactorial, standard coefficient alpha (Cronbach, 1951), coefficient alpha for ordinal data (Chalmers, 2018; Zumbo and Kroc, 2019), and marginal reliabilities (Chalmers, 2012) all provide appropriate reliability estimates. For the final 83-item scale, the Cronbach alphas for the 83-item scale are in Table 3 (lower right): *α* = 0.88 for the overall scale, *αs* = 0.70 to 0.75 for three subscales, and *α* = 0.56 for Connecting. Ordinal alphas were somewhat higher at *α* = 0.93 overall, and for Perceiving, Connecting, Understanding, and Managing, 0.82, 0.68, 0.85, and 0.83, respectively. Lastly, the marginal reliabilities were 0.89, 0.73, 0.62, 0.77, and 0.73, respectively. The lower internal consistency of the Connecting domain reflected a lack of a sufficient number of good items for that area from the Pilot Study (Study 3) forward, although the factor clearly emerged. As indicated in the Discussion, it is retained owing to evidence here and elsewhere that it is a replicable factor of theoretical importance.

#### 6.3.8 Did the MSCEIT 2 measure high ability EI with sufficient precision?

**Figure 4:**
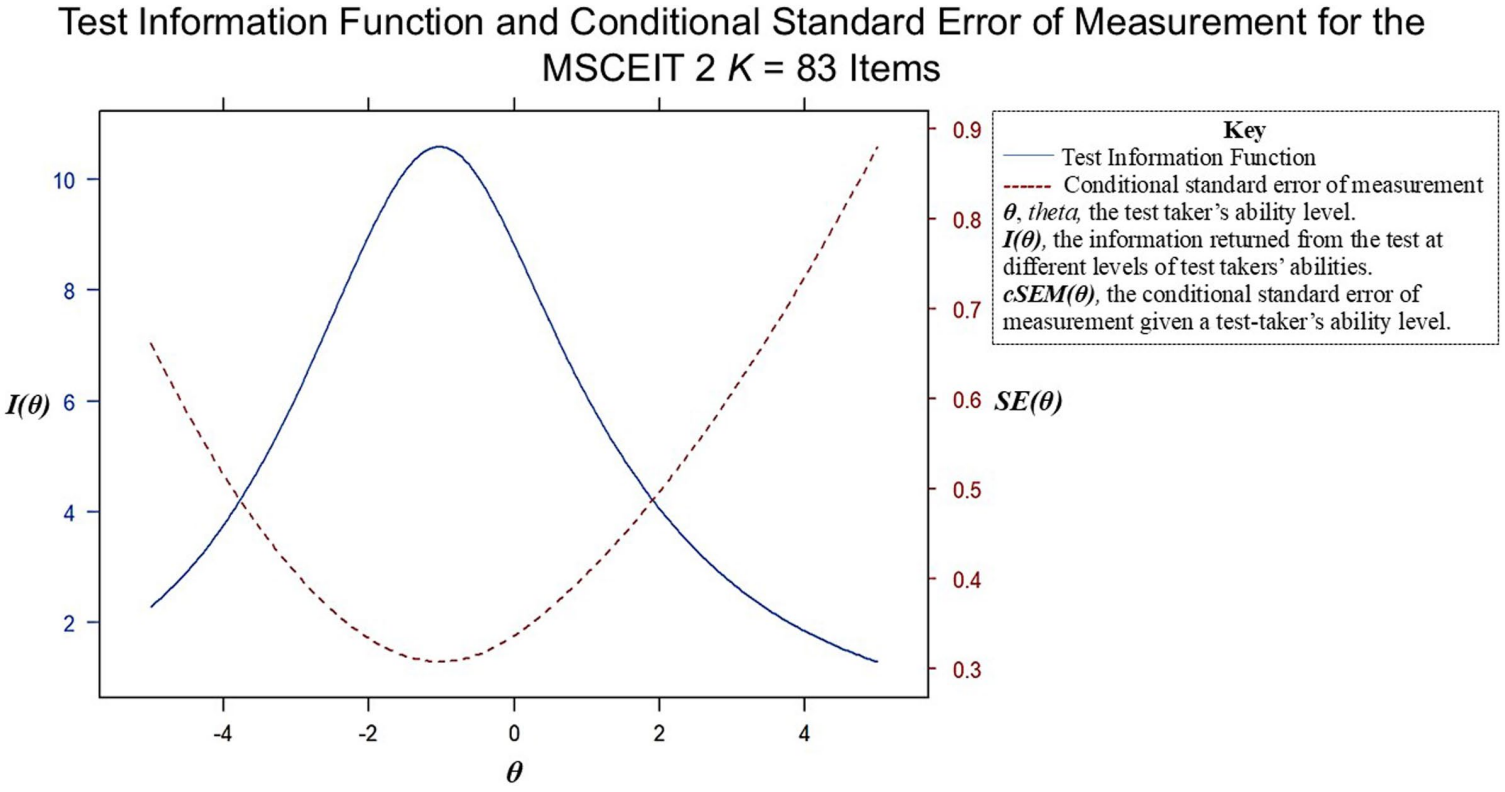
Test information function (TIF) and conditional standard error of measurement (cSEM) for the MSCEIT 2. The TIF indicates the degree of test information returned, and the cSEM the precision of measurement at different levels of test-taker ability.

We used a two-parameter IRT model at several stages of test development, removing items with *b* parameters indicating they were redundant with others in measuring lower EI ability, and preserving items that measured higher levels of ability. The Test Information Function for the 83-item version of the test is shown in Figure 4. A test-taker’s scaled score of 130 points (on a scale of *M* = 100, *SD* = 15) would represent an ability (i.e., theta) level of *θ* = 2, two standard deviations above the mean. Such a score would have a conditional standard error of measurement (CSEM) of approximately *0.*5, yielding a 95% confidence interval of 130 +/− 14.7 (14.7 = 0.5 × 1.96 × 15), meaning that their true ability would fall between 115.3 to 147.3, 95% of the time. The CSEM at an EI score of 85, by comparison, would be about +/− 8.8 (8.8 = 0.30 × 1.96 × 15), or a range between 76 and 94, 95% of the time. The information functions and associated CSEM values indicate that the precision of measurement remained better for the below-average group relative to the above-average group, despite our attempt to equalize precision across ability levels. This pattern may be a characteristic of people-centered intelligence scales more generally (e.g., Mayer et al., 2019). That said, the precision for high-ability test-takers is reasonably informative at higher levels where, for example, scores of approximately 115 and above represent above-average performance 95% of the time, with higher scores reflecting the increasing probability the test-taker is high in ability.

### 6.4 Discussion of the normative sample study

Study 4, of the normative sample, indicated the viability of the four-factor model of EI. Broad intelligences generally correlate with one another at about the *r* = 0.60 level, although similar broad intelligences such as mathematical and spatial correlate higher still, and dissimilar broad intelligences, such as the spatial and emotional, hardly correlate at all (Bryan and Mayer, 2020, 2021). All-told, the four domains should correlate something above *r* = 0.60 but less than *r* = 0.95 (to indicate some distinction between them). The estimated correlations among Connecting, Understanding, and Managing Emotions were, in fact, between *r* = 0.77 and 0.91, supporting their status as subsidiary factors of EI. Perceiving Emotions, however, was lower, at *r* = 0.52 to 0.57 raising whether visual stimuli may call on skills beyond the focal emotion perception skills targeted here.

## 7 Study 5: MSCEIT-MSCEIT 2 correlations

### 7.1 Overview

Study 5 was conducted to examine test continuity between the original and revised tests. Recall that the MSCEIT 2 was substantially revised relative to the original MSCEIT, with virtually no items identical across forms. By comparison, for example, the WAIS-IV retained 12 subtests from the WAIS-III and those subtests often repeated items; for example, about 33% of WAIS Similarities and 70% of Vocabulary items were repeated, with a resulting *r* = 0.94 between forms (Wechsler, 2008). Because of our evolving theoretical understanding of EI, and the consequent differences between the MSCEIT and MSCEIT 2 in their item groups, scoring methods, and response formats, their correlation was likely to be more modest, perhaps between *r* = 0.60 and 0.90. Regarding the subscales, the relatively similar formats of the Understanding and (to a lesser degree) Managing domains of the MSCEIT and MSCEIT 2 should exhibit higher correlations across forms than the more substantially changed domains of Perceiving and Connecting.

### 7.2 Participants, method, and measures

The MSCEIT and MSCEIT 2 were administered on *Prolific* (e.g., Palan and Schitter, 2018) to an initial sample of *N* = 229 of whom 8 were screened out due to inattention. The final *N* = 221 participants consisted of 98 female, 122 male, and 1 “other” participant of whom 10.0% were Asian, 6.8% Black, 10.9% Hispanic, 71.0% White, and 1.4% Other/Unspecified. The sample had a high education level overall (93% some college or higher). The two tests were administered in counterbalanced order approximately 2 weeks apart.

### 7.3 Results and discussion

For Study 5, scores for both the MSCEIT 2 and MSCEIT were calculated with reference to their normative samples, which were scaled to an overall *M* = 100 and *S* = 15. The participants scored ten points higher on the MSCEIT 2 than on the MSCEIT, *M* = 114 and 104, respectively, with *S* of 11.9 and 12.8, suggesting the MSCEIT 2 may yield higher scores than the original MSCEIT. The Prolific sample exhibited a restricted score range, perhaps owing to their relatively high education level. Corrections for restriction of range (Dahlke and Wiernik, 2019) as well as for unreliability were applied.

A moderate correlation had been expected between the MSCEIT and the MSCEIT 2 given the changes made to the MSCEIT 2, including the addition of new question types and all new items, new response scales for three of the domains, and a new scoring method. The correlations between the MSCEIT and MSCEIT 2 overall and domains scores are indicated in Table 5. The overall correlation of the MSCEIT and MSCEIT 2 was *r* = 0.69, corrected for unreliability and restriction of range. The corresponding MSCEIT and MSCEIT 2 domains generally correlated with one another. The Understanding domain was most similar and correlated *r* = 0.93 across the original MSCEIT and MSCEIT 2. Connecting, which was substantially different from Facilitation on the original test, correlated just *r* = 0.29. Collectively, these findings suggested that the variance of the original MSCEIT that reflected actual EI had been maintained, clarified and, we believe, incremented in the new form.

**Table 5:** Corrected^a^ correlations between the MSCEIT and MSCEIT 2 scales (*N* = 221).

		MSCEIT 2				
		Overall	Perceive	Connect	Understand	Manage
Original MSCEIT	Overall	0.69	0.66	0.44	0.60	0.66
	Perceive	0.38	0.41	0.26	0.21	0.33
	Facilitate	0.50	0.52	0.29	0.41	0.46
	Understand	0.91	0.77	0.71	0.93	0.87
	Manage	0.53	0.55	0.19	0.49	0.61

a Correlations were corrected for attenuation due to unreliability and restriction of range.

## 8 General discussion

Over the past two decades, discussions in the emotions and intelligence literatures, as well as empirical studies employing the original MSCEIT and other EI ability scales, have led to revisions in our thinking about EI. We now view ability EI as a broad intelligence that fits reasonably well within the Cattell-Horn-Carroll (CHC) three-stratum model of intelligence. We regard veridical scoring—item development using literature reviews and having experts employ these scoring manuals to assign scores and remove problematic items—as an optimal scoring process. We regard EI abilities as consisting of more measurable skills than before—which can affect a given measure’s factor structure. The MSCEIT 2 implemented changes that reflected these evolving theoretical views—using more item sets and veridical scoring, more consistent response alternatives, and also employing more engaging question types and requiring less time to administer.

Validity evidence from the MSCEIT 2’s content stemmed from its close adherence to the ability model of EI (Mayer and Salovey, 1997; Mayer et al., 2016; Mayer et al., 2024) which specifies Perceiving, Connecting (formerly, Facilitating), Understanding, and Managing domains of problem-solving about emotions. Evidence for the test’s validity from response processes is based on general agreement that intelligence is optimally measured by observing how people solve mental problems and evaluating their responses on a criterion of correctness (Sheldon et al., 2014; Neubauer and Hofer, 2021; Mayer and Bryan, 2024). Veridical scoring ensures that scoring approaches are similar to those used in other ability assessments.

In the Pilot and Normative sample studies (Studies 3 and 4) we examined validity evidence for the MSCEIT 2’s test structure. There was considerable continuity across the two studies with the exception that the 1- and 2-factor solutions, which fit the Study 3 Pilot data reasonably well, failed to fit the Study 4 Normative sample. For both the Study 3 and 4 data, however, a 4-factor model corresponding to the four-domain ability model fit the test better than the alternatives. A hierarchical model that included an Overall EI fit equally well.

Based on these findings, an 83-item test was created that provided scores for overall EI and each of the four domains. The reliability estimates varied somewhat depending upon the formula used (i.e., alpha, ordinal alpha, or marginal reliabilities). The alpha reliabilities were very good for the overall test (*α* = 0.88), which compares favorably to other intelligence tests (Charter, 2003; Oosterwijk et al., 2019). They were respectable for Perceiving (0.70) and for Understanding and Managing (0.75), but markedly lower for Connecting Emotions (0.56); we note that the ordinal and marginal reliabilities for Connecting were somewhat higher at 0.68 and 0.62, respectively. The precision of the overall EI score was better for lower scorers but still informative at higher ability levels. Connecting Emotions scores are a matter of interest but should not be regarded as diagnostic at the individual level short of extreme score differences.

The final version of the MSCEIT 2 consists of 83 scored items, embedded amidst 94 items including an additional 5 unscored items, 5 attention checks, and one self-report item asking about performance. The 94-item version compared to the 141-item original MSCEIT represents a 1/3^rd^ reduction in length.

### 8.1 Limitations and concerns

#### 8.1.1 Remaining issues

The construction of the MSCEIT 2 involved applying the EI construct to item development, data collection, statistical analyses, and studies of the relationship between the test and its earlier version. These involve interrelated processes: changes in item composition, for example, affect the items’ statistical performance. Each area of test development has its own constraints, and compromises are sometimes needed over the test-development process. At the outset, we hoped to improve and revise many aspects of the MSCEIT and although we have succeeded in some areas such as test friendliness, item scoring, and test structure, we also ended up compromising in other areas such as domain reliability, particularly for the Connecting domain. We discuss these issues next.

#### 8.1.2 Should the Connecting domain (formerly, Facilitation) be removed?

A reasonable concern can be raised over whether the Connecting Emotions domain score should be dropped given its reliability is below typically-acceptable thresholds. We have retained it because its continued inclusion slightly raises the reliability of the overall test, and, additionally, the preponderance of factor analytic evidence across similar scales indicates that there is a genuine ability being assessed (i.e., Mayer et al., 2024). The scale is, in this sense, more than a mere placeholder but less than an ideal measure. And *not* to measure this domain could overlook or underestimate some test-takers’ abilities. That said, test administrators have the option to select the domains they wish to employ when using the MSCEIT 2, which was not possible with the original test.

Returning to the idea that the Connecting domain may tap a unique ability, one of the original ideas of the domain (formerly “Facilitation”) was that emotions assist thinking and perhaps contribute to creativity (Taylor, 2017; Holm-Hadulla et al., 2021; Greenwood et al., 2022). Connecting Emotions captures this aspect: For some forms of artistic creation, for example, artists must find the connection between different aspects of emotional expression. A musician, for instance, might connect sad emotions to the low energy that often accompanies sad states, and then express the torpor in a composition with a slowed-down, meandering melody; a visual artist might do so by using the muted colors of inclement, overcast weather. Similarly, an acting coach who connects shock to the expression that shock “sucks the air out of you,” might direct an actor trying to capture the emotion to exhale quickly and then try to catch their breath. Beyond the arts, such processes may facilitate finding and implementing solutions about how to behave in interpersonal situations, communicating with others more empathically, and enhancing decision-making more generally. Although the second domain remains a work in progress and scores on the Connecting scale should be treated cautiously, it seems promising.

#### 8.1.3 The 4-factor model as a continued guide

The estimated correlation of *r* = 0.91 between Understanding and Managing domains in the simple structure factor model (Figure 3, left) raises the issue of whether merging those two would be best. No other between-domain correlation rose above *r* = 0.86 in the final 4-factor model. It seems reasonable to accept the two abilities as highly related but distinct because distinct Understanding and Managing factors have been found across nearly all broad-spectrum EI tests (Mayer et al., 2024). At the low end of the relations, the correlations between Perceiving and the other domains, in the *r* = 0.50 range, could argue that it is a distinct broad intelligence, but before drawing such a conclusion more evidence from other tests such as the Geneva Emotional Competence scale would be helpful (e.g., Schlegel and Mortillaro, 2019). For now, the four-domain approach seems prudent as a guide.

#### 8.1.4 Is EI best considered a single factor?

A further question concerning the high correlations among (most) domain scores is whether EI is best considered a single factor. For many purposes, this is a reasonable approach. Yet dividing EI into domains when interpreting a person’s performance may help guide educational and training approaches and further clarify the type of reasoning that people carry out; that said, interpreting differences in scale scores should be approached cautiously, understanding that those differences will need to be substantial to be interpreted. As reported in these studies, the data do support a 4-factor model that yields 4 domain scores.

### 8.2 Statement of generalizability

The normative sample employed here reflected the populations of the U.S. and Canada, and were representative of age, gender, education, and four ethnic groups of those nations. The United States and Canada are, however, relatively wealthy Western nations, and the test characteristics will require some modifications when adapted to different nations and languages, as had been the case with the earlier MSCEIT. The generalizability of the measure beyond such cultures is largely unknown. Cognition and emotional expression vary with both literacy and culture and therefore generalizations to non-Western groups should be approached cautiously (e.g., Ong, 2002; Henrich et al., 2010; Han et al., 2019; Briceño et al., 2023).

### 8.3 The advantages of ability measures and transition to the MSCEIT 2

In keeping with our remarks on the importance of employing ability-based measures of emotional intelligence, the MSCEIT 2 was uncorrelated with test-takers’ own estimates of their skills (*r* = −0.07)—similar to that found in other studies (Sheldon et al., 2014; Neubauer and Hofer, 2021). The MSCEIT 2 did, however, correlate with the original MSCEIT, *r* = 0.69, indicating it will be similarly or—we expect—more highly correlated with key criteria than the original owing to its improved scoring and mitigation of response scale artifacts, as well as its briefer, more engaging nature. The measure also was designed throughout to be a fair test across groups by including content that was diverse and by removing items that appeared statistically different across demographic groups (see test manual for additional details, Mayer et al., 2025).

## 9 Conclusion

This article described how the evolving understanding of EI over the past 35 years influenced the development of the MSCEIT 2. The MSCEIT 2 has support for its validity based on its item content, the response processes it elicits from test-takers, and the factor structure on which its overall score and scale scores are based, as well as its relation to the original MSCEIT. The test and its scale reliabilities were generally good with the exception of the Connecting Emotions scale. Our hope is that the MSCEIT 2 will serve as an up-to-date and improved measure for research and assessment of emotional intelligence.

The importance of ability-based measures of EI should not be underestimated. They operationalize key aspects of how people process emotions, and individual differences in such processing. They have contributed to the understanding of people-centered intelligences such as personal and social intelligence more generally (Bryan and Mayer, 2021), they make meaningful incremental improvements in predictions of both academic achievement and on-the-job performance (Joseph and Newman, 2010; O’Boyle et al., 2011; MacCann et al., 2020) and are related to lower psychopathology in several areas (Ermer et al., 2012; DeTore et al., 2018; Gómez-Leal et al., 2021), as well as better social relations (Brackett et al., 2004; Brackett et al., 2006). Such findings argue for the continued measurement and use of the construct in psychological research.

## Data Availability

The datasets presented in this article are not readily available because of “critical concerns regarding…test security” (see Ethics Code Standard 9.11 at https://www.apa.org/ethics/code) including the potential invalidation of test administrations, and the authors’ contractual and associated ethical obligations. Although the raw test data is not available, details about many analyses reported here are available in the accompanying Technical Supplement (Mayer et al., 2024). The manuscript meets Level 1 standards of the Transparency and Openness (TOP) Guidelines. Requests to access the datasets should be directed to www.mhs.com.

## References

[ref1] Ashkanasy N. M. Daus C. S. (2005). Rumors of the death of emotional intelligence in organizational behavior are vastly exaggerated. J. Organ. Behav. 26, 441–452. doi: 10.1002/job.320

[ref2] Brackett M. A. Mayer J. D. Warner R. M. (2004). Emotional intelligence and its relation to everyday behaviour. Personal. Individ. Differ. 36, 1387–1402. doi: 10.1016/S0191-8869(03)00236-8

[ref3] Brackett M. A. Rivers S. E. Shiffman S. Lerner N. Salovey P. (2006). Relating emotional abilities to social functioning: a comparison of self-report and performance measures of emotional intelligence. J. Pers. Soc. Psychol. 91, 780–795. doi: 10.1037/0022-3514.91.4.780, PMID: 17014299

[ref4] Briceño E. M. Arce Rentería M. Gross A. L. Jones R. N. Gonzalez C. Wong R.. (2023). A cultural neuropsychological approach to harmonization of cognitive data across culturally and linguistically diverse older adult populations. Neuropsychology 37, 247–257. doi: 10.1037/neu0000816, PMID: 35482625 PMC9639608

[ref5] Bryan V. M. Mayer J. D. (2020). A meta-analysis of the correlations among broad intelligences: understanding their relations. Intelligence 81:101469. doi: 10.1016/j.intell.2020.101469

[ref6] Bryan V. M. Mayer J. D. (2021). Are people-centered intelligences psychometrically distinct from thing-centered intelligences? A meta-analysis. J. Intelligence 9:48. doi: 10.3390/jintelligence9040048, PMID: 34698222 PMC8544294

[ref7] Burisch M. (1984). You don’t always get what you pay for: measuring depression with short and simple versus long and sophisticated scales. J. Res. Pers. 18, 81–98. doi: 10.1016/0092-6566(84)90040-0

[ref8] Carroll J. B. (1993). Human cognitive abilities: A survey of factor-analytic studies. New York, NY: Cambridge University Press.

[ref9] Chalmers R. P. (2012). Mirt: a multidimensional item response theory package for the R environment. J. Stat. Softw. 48, 1–29. doi: 10.18637/jss.v048.i06

[ref10] Chalmers R. P. (2018). On misconceptions and the limited usefulness of ordinal alpha. Educ. Psychol. Meas. 78, 1056–1071. doi: 10.1177/0013164417727036, PMID: 30559513 PMC6293415

[ref11] Chandler J. Rosenzweig C. Moss A. J. Robinson J. Litman L. (2019). Online panels in social science research: expanding sampling methods beyond mechanical Turk. Behav. Res. Methods 51, 2022–2038. doi: 10.3758/s13428-019-01273-7, PMID: 31512174 PMC6797699

[ref12] Charter R. A. (2003). A breakdown of reliability coefficients by test type and reliability method, and the clinical implications of low reliability. J. Gen. Psychol. 130, 290–304. doi: 10.1080/00221300309601160, PMID: 12926514

[ref13] Cherniss C. (2010). Emotional intelligence: new insights and further clarifications. Indust. Organ. Psychol. 3, 183–191. doi: 10.1111/j.1754-9434.2010.01222.x

[ref14] Chu J. Qaisar S. Shah Z. Jalil A. (2021). Attention or distraction? The impact of mobile phone on users’ psychological well-being. Front. Psychol. 12:612127. doi: 10.3389/fpsyg.2021.612127, PMID: 33959065 PMC8093572

[ref15] Cliff N. (1993). Dominance statistics: ordinal analyses to answer ordinal questions. Psychol. Bull. 114, 494–509. doi: 10.1037/0033-2909.114.3.494

[ref16] Credé M. Harms P. Niehorster S. Gaye-Valentine A. (2012). An evaluation of the consequences of using short measures of the big five personality traits. J. Pers. Soc. Psychol. 102, 874–888. doi: 10.1037/a0027403, PMID: 22352328

[ref17] Cronbach L. J. (1951). Coefficient alpha and the internal structure of tests. Psychometrika 16, 297–334. doi: 10.1007/BF02310555, PMID: 40103751

[ref18] Cronbach L. J. Meehl P. E. (1955). Construct validity in psychological tests. Psychol. Bull. 52, 281–302. doi: 10.1037/h0040957, PMID: 13245896

[ref19] Dahlke J. A. Wiernik B. M. (2019). Psychmeta: an R package for psychometric meta-analysis. Appl. Psychol. Meas. 43, 415–416. doi: 10.1177/0146621618795933, PMID: 31235986 PMC6572911

[ref20] Darwin C. (1872/2024). The descent of man. London, England: Penguin Classics.

[ref21] Davies M. Stankov L. Roberts R. D. (1998). Emotional intelligence: in search of an elusive construct. J. Pers. Soc. Psychol. 75, 989–1015. doi: 10.1037/0022-3514.75.4.989, PMID: 9825531

[ref22] de Vries R. E. (2013). The 24-item brief HEXACO inventory (BHI). J. Res. Pers. 47, 871–880. doi: 10.1016/j.jrp.2013.09.003

[ref23] DeTore N. R. Mueser K. T. McGurk S. R. (2018). What does the managing emotions branch of the MSCEIT add to the MATRICS consensus cognitive battery?: schizophrenia research. Schizophr. Res. 197, 414–420. doi: 10.1016/j.schres.2018.02.018, PMID: 29486955 PMC13181848

[ref24] Ekman P. (1993). Facial expression and emotion. Am. Psychol. 48, 384–392. doi: 10.1037/0003-066X.48.4.384 8512154

[ref25] Ermer E. Kahn R. E. Salovey P. Kiehl K. A. (2012). Emotional intelligence in incarcerated men with psychopathic traits. J. Pers. Soc. Psychol. 103, 194–204. doi: 10.1037/a0027328, PMID: 22329657 PMC3378803

[ref26] Evans T. R. Hughes D. J. Steptoe-Warren G. (2020). A conceptual replication of emotional intelligence as a second-stratum factor of intelligence. Emotion 20, 507–512. doi: 10.1037/emo0000569, PMID: 30730169

[ref27] Fan H. Jackson T. Yang X. Tang W. Zhang J. (2010). The factor structure of the Mayer–Salovey–Caruso emotional intelligence test V 2.0 (MSCEIT): a meta-analytic structural equation modeling approach. Personal. Individ. Differ. 48, 781–785. doi: 10.1016/j.paid.2010.02.004

[ref28] Firth J. Torous J. Stubbs B. Firth J. A. Steiner G. Z. Smith L.. (2019). The “online brain”: how the internet may be changing our cognition. World Psychiatry 18, 119–129. doi: 10.1002/wps.20617, PMID: 31059635 PMC6502424

[ref29] Flora D. B. Flake J. K. (2017). The purpose and practice of exploratory and confirmatory factor analysis in psychological research: decisions for scale development and validation. Canadian J. Behav. Sci. 49, 78–88. doi: 10.1037/cbs0000069

[ref30] Føllesdal H. Hagtvet K. A. (2009). Emotional intelligence: the MSCEIT from the perspective of generalizability theory. Intelligence 37, 94–105. doi: 10.1016/j.intell.2008.08.005

[ref31] Gómez-Leal R. Megías-Robles A. Sánchez-López M. T. Fernández-Berrocal P. (2021). Psychopathic traits and ability emotional intelligence in incarcerated males. Eur. J. Psychol. Appl. Legal Context 13, 79–86. doi: 10.5093/ejpalc2021a8

[ref32] Gorsuch R. L. (2015). Factor analysis. New York, NY US: Routledge.

[ref33] Greenwood T. A. Chow L. J. Gur R. C. Kelsoe J. R. (2022). Bipolar spectrum traits and the space between madness and genius: the muse is in the dose. J. Psychiatr. Res. 153, 149–158. doi: 10.1016/j.jpsychires.2022.07.009, PMID: 35816974

[ref34] Han K. Colarelli S. M. Weed N. C. (2019). Methodological and statistical advances in the consideration of cultural diversity in assessment: a critical review of group classification and measurement invariance testing. Psychol. Assess. 31, 1481–1496. doi: 10.1037/pas0000731, PMID: 31763873

[ref35] Henrich J. Heine S. J. Norenzayan A. (2010). Beyond WEIRD: towards a broad-based behavioral science. Behav. Brain Sci. 33, 111–135. doi: 10.1017/S0140525X10000725, PMID: 40078805

[ref36] Hertel J. Schütz A. Lammers C.-H. (2009). Emotional intelligence and mental disorder. J. Clin. Psychol. 65, 942–954. doi: 10.1002/jclp.20597, PMID: 19437504

[ref37] Holm-Hadulla R. M. Hofmann F. H. Sperth M. Mayer C. H. (2021). Creativity and psychopathology: an interdisciplinary view. Psychopathology 54, 39–46. doi: 10.1159/000511981, PMID: 33326984

[ref38] Hubley A. M. Zumbo B. D. (2017). “Response processes in the context of validity: setting the stage” in Understanding and investigating response processes in validation research. eds. Zumbo B. D. Hubley A. M. (Cham: Springer International Publishing), 1–12.

[ref39] Joint Committee (2014). Standards for educational and psychological testing. Washington, DC US: American Psychological Association.

[ref40] Joseph D. L. Jin J. Newman D. A. O'Boyle E. H. (2015). Why does self-reported emotional intelligence predict job performance? A meta-analytic investigation of mixed EI. J. Appl. Psychol. 100, 298–342. doi: 10.1037/a0037681, PMID: 25243996

[ref41] Joseph D. L. Newman D. A. (2010). Emotional intelligence: an integrative meta-analysis and cascading model. J. Appl. Psychol. 95, 54–78. doi: 10.1037/a0017286, PMID: 20085406

[ref42] Kruyen P. M. Emons W. H. M. Sijtsma K. (2013). On the shortcomings of shortened tests: a literature review. Int. J. Test. 13, 223–248. doi: 10.1080/15305058.2012.703734

[ref43] Legree P. J. Psotka J. Robbins J. Roberts R. D. Putka D. J. Mullins H. M. (2014). Profile similarity metrics as an alternate framework to score rating-based tests: MSCEIT reanalyses. Intelligence 47, 159–174. doi: 10.1016/j.intell.2014.09.005

[ref44] Lin I. Pothera M. (2019). Beta study research design: MSCEIT 2 user experience research. Toronto, ON: MultiHealth Systems.

[ref45] Little T. D. Cunningham W. A. Shahar G. Widaman K. F. (2002). To parcel or not to parcel: exploring the question, weighing the merits. Struct. Equ. Model. 9, 151–173. doi: 10.1207/S15328007SEM0902_1

[ref46] Little T. D. Rhemtulla M. Gibson K. Schoemann A. M. (2013). Why the items versus parcels controversy needn’t be one. Psychol. Methods 18, 285–300. doi: 10.1037/a0033266, PMID: 23834418 PMC3909043

[ref47] Locke E. A. (2005). Why emotional intelligence is an invalid concept. J. Organ. Behav. 26, 425–431. doi: 10.1002/job.318

[ref48] Loevinger J. (1947). A systematic approach to the construction and evaluation of tests of ability. Psychol. Monogr. 61, 1–49. doi: 10.1037/h0093565

[ref49] Lopes P. N. Salovey P. Côté S. Beers M. (2005). Emotion regulation abilities and the quality of social interaction. Emotion 5, 113–118. doi: 10.1037/1528-3542.5.1.113, PMID: 15755224

[ref50] MacCann C. Jiang Y. Brown L. E. R. Double K. S. Bucich M. Minbashian A. (2020). Emotional intelligence predicts academic performance: a meta-analysis. Psychol. Bull. 146, 150–186. doi: 10.1037/bul0000219, PMID: 31829667

[ref51] MacCann C. Joseph D. L. Newman D. A. Roberts R. D. (2014). Emotional intelligence is a second-stratum factor of intelligence: evidence from hierarchical and bifactor models. Emotion 14, 358–374. doi: 10.1037/a0034755, PMID: 24341786

[ref52] MacCann C. Roberts R. D. (2008). New paradigms for assessing emotional intelligence: theory and data. Emotion 8, 540–551. doi: 10.1037/a0012746, PMID: 18729584

[ref53] Marder S. R. Fenton W. Youens K. (2004). Schizophrenia, IX: cognition in schizophrenia—the MATRICS initiative. Am. J. Psychiatry 161:25. doi: 10.1176/appi.ajp.161.1.25 14702244

[ref54] Mayer J. D. Bryan V. M. (2024). On personality measures and their data: a classification of measurement approaches and their recommended uses. Personal. Soc. Psychol. Rev. 28, 325–345. doi: 10.1177/10888683231222519, PMID: 38314773

[ref9001] Mayer J. D. Caruso D. R. (2025). Emotional intelligence and socioemotional attributes: distinct constructs and their uses. Consult. Psychol. J. 1–19. doi: 10.1037/cpb0000293

[ref55] Mayer J. D. Caruso D. R. Panter A. T. (2019). Advancing the measurement of personal intelligence with the test of personal intelligence, version 5 (TOPI 5). J. Intelligence 7, 1–17. doi: 10.3390/jintelligence7010004, PMID: 31162383 PMC6526446

[ref56] Mayer J. D. Caruso D. R. Salovey P. (1999). Emotional intelligence meets traditional standards for an intelligence. Intelligence 27, 267–298. doi: 10.1016/S0160-2896(99)00016-1

[ref57] Mayer J. D. Caruso D. R. Salovey P. (2016). The ability model of emotional intelligence: principles and updates. Emot. Rev. 8, 290–300. doi: 10.1177/1754073916639667, PMID: 40103853

[ref58] Mayer J. D. Caruso D. R. Salovey P. (2024) Technical supplement for ‘measuring emotional intelligence with the MSCEIT 2’. Available online at: https://osf.io/nmp68/ (Accessed December 4, 2024).

[ref59] Mayer J. D. Caruso D. R. Salovey P. (2025). Mayer-Salovey-Caruso emotional intelligence test 2 (MSCEIT 2) User’s manual. Toronto, ON: MHS.

[ref60] Mayer J. D. Caruso D. R. Sitarenios G. Escobar M. R. (2024). How many emotional intelligence abilities are there? An examination of four measures of emotional intelligence. Personal. Individ. Differ. 219:112468. doi: 10.1016/j.paid.2023.112468

[ref61] Mayer J. D. DiPaolo M. Salovey P. (1990). Perceiving affective content in ambiguous visual stimuli: a component of emotional intelligence. J. Pers. Assess. 54, 772–781. doi: 10.1080/00223891.1990.9674037, PMID: 2348356

[ref62] Mayer J. D. Geher G. (1996). Emotional intelligence and the identification of emotion. Intelligence 22, 89–113. doi: 10.1016/S0160-2896(96)90011-2

[ref63] Mayer J. D. Salovey P. (1997). “What is emotional intelligence?” in Emotional development and emotional intelligence: Educational implications. ed. Sluyter D. J. (New York, NY: Basic Books), 3–34.

[ref64] Mayer J. D. Salovey P. Caruso D. R. (2002). Mayer-Salovey-Caruso emotional intelligence test: (MSCEIT): User’s manual. Toronto, ON: MHS.

[ref65] Mayer J. D. Salovey P. Caruso D. R. (2014). Mayer-Salovey-Caruso emotional intelligence test: Youth research version, MSCEIT: YRV. Toronto, ON: MHS.

[ref66] Mayer J. D. Salovey P. Caruso D. R. Sitarenios G. (2001). Emotional intelligence as a standard intelligence. Emotion 1, 232–242. doi: 10.1037/1528-3542.1.3.232 12934682

[ref67] Mayer J. D. Salovey P. Caruso D. R. Sitarenios G. (2003). Measuring emotional intelligence with the MSCEIT V2.0. Emotion 3, 97–105. doi: 10.1037/1528-3542.3.1.97, PMID: 12899321

[ref68] McCready W. C. (1996). “Applying sampling procedures” in The psychology research handbook: A guide for graduate students and research assistants. eds. Leong F. T. L. Austin J. T. (Thousand Oaks, CA: Sage Publications, Inc), 98–110.

[ref69] McGrew K. S. (2009). CHC theory and the human cognitive abilities project: standing on the shoulders of the giants of psychometric intelligence research. Intelligence 37, 1–10. doi: 10.1016/j.intell.2008.08.004

[ref70] McNemar Q. (1940). Sampling in psychological research. Psychol. Bull. 37, 331–365. doi: 10.1037/h0063476

[ref71] Mendoza J. S. Pody B. C. Lee S. Kim M. McDonough I. M. (2018). The effect of cellphones on attention and learning: the influences of time, distraction, and nomophobia. Comput. Hum. Behav. 86, 52–60. doi: 10.1016/j.chb.2018.04.027

[ref72] Messick S. (1995). Validity of psychological assessment: validation of inferences from persons’ responses and performances as scientific inquiry into score meaning. Am. Psychol. 50, 741–749. doi: 10.1037/0003-066X.50.9.741

[ref9002] Muthén L. K. Muthén B. O. (2017). Mplus user’s guide (Eighth). Muthén & Muthén.

[ref73] Mweshi G. K. Sakyi K. (2020). Application of sampling methods for the research design. Arch. Bus. Res. 8, 180–193. doi: 10.14738/abr.811.9042

[ref74] Neubauer A. C. Hofer G. (2021). Self-estimates of abilities are a better reflection of individuals’ personality traits than of their abilities and are also strong predictors of professional interests. Personal. Individ. Differ. 169, 109850–109815. doi: 10.1016/j.paid.2020.109850, PMID: 40104671

[ref75] Neuhäuser M. Lösch C. Jöckel K.-H. (2007). The Chen-Luo test in case of heteroscedasticity. Comput. Statist. 51:5055.

[ref76] Nunnally J. C. (1967). Psychometric theory. New York, NY: McGraw-Hill.

[ref77] O’Boyle E. H. Humphrey R. H. Pollack J. M. Hawver T. H. Story P. A. (2011). The relation between emotional intelligence and job performance: a meta-analysis. J. Organ. Behav. 32, 788–818. doi: 10.1002/job.714

[ref78] Ong W. (2002). “Orality and literacy: writing restructures consciousness” in The book history reader. eds. Finkelstein D. McCleery A. (London, England: Routledge), 105–117.

[ref79] Oosterwijk P. R. van der Ark L. A. Sijtsma K. (2019). Using confidence intervals for assessing reliability of real tests. Assessment 26, 1207–1216. doi: 10.1177/1073191117737375 29084436

[ref80] Palan S. Schitter C. (2018). Prolific.ac—a subject pool for online experiments. J. Behav. Exp. Financ. 17, 22–27. doi: 10.1016/j.jbef.2017.12.004

[ref81] Palmer B. R. Gignac G. Manocha R. Stough C. (2005). A psychometric evaluation of the Mayer-Salovey-Caruso emotional intelligence test version 2.0. Intelligence 33, 285–305. doi: 10.1016/j.intell.2004.11.003

[ref82] Plutchik R. (2001). The nature of emotions. Am. Sci. 89, 344–350. doi: 10.1511/2001.28.344

[ref83] Roberts R. D. Zeidner M. Matthews G. (2001). Does emotional intelligence meet traditional standards for an intelligence? Some new data and conclusions. Emotion 1, 196–231. doi: 10.1037/1528-3542.1.3.196, PMID: 12934681

[ref84] Salovey P. Mayer J. D. (1990). Emotional intelligence. Imagin. Cogn. Pers. 9, 185–211. doi: 10.2190/DUGG-P24E-52WK-6CDG

[ref85] Schlegel K. Grandjean D. Scherer K. R. (2013). Constructs of social and emotional effectiveness: different labels, same content? J. Res. Pers. 47, 249–253. doi: 10.1016/j.jrp.2013.02.005

[ref86] Schlegel K. Mortillaro M. (2019). The Geneva emotional competence test (GECo): an ability measure of workplace emotional intelligence. J. Appl. Psychol. 104, 559–580. doi: 10.1037/apl0000365, PMID: 30346195

[ref87] Schneider W. J. Mayer J. D. Newman D. A. (2016). Integrating hot and cool intelligences: thinking broadly about broad abilities. J. Intelligence 4, 1–25. doi: 10.3390/jintelligence4010001

[ref88] Shaffer L. F. Gilmer B. Von H. Schoen M. (1940). Psychology. New York, NY: Harper.

[ref89] Sheldon O. J. Dunning D. Ames D. R. (2014). Emotionally unskilled, unaware, and uninterested in learning more: reactions to feedback about deficits in emotional intelligence. J. Appl. Psychol. 99, 125–137. doi: 10.1037/a0034138, PMID: 23957689

[ref90] Statistics Canada (2023) ‘Census profile. 2021 census of population. Statistics Canada catalogue no. 98-316-X2021001.’ Statistics Canada. Available online at: https://www12.statcan.gc.ca/census-recensement/2021/dp-pd/prof/index.cfm?Lang=E (accessed April 30, 2024).

[ref91] Taylor C. L. (2017). Creativity and mood disorder: a systematic review and meta-analysis. Perspect. Psychol. Sci. 12, 1040–1076. doi: 10.1177/1745691617699653, PMID: 28934560

[ref92] U.S. Census Bureau (2023) ‘Selected population profile in the United States. American community survey, ACS 1-year estimates selected population profiles.’

[ref93] Wang S. De Boeck P. Yotebieng M. (2023). Heywood cases in unidimensional factor models and item response models for binary data. Appl. Psychol. Meas. 47, 141–154. doi: 10.1177/01466216231151701, PMID: 36875295 PMC9979198

[ref94] Wechsler D. (2008). WAIS-IV: Wechsler adult intelligence scale: technical and interpretive manual. Fourth Ed. San Antonio, TX: The Psychological Corporation.

[ref95] Young P. T. (1936). Motivation of behavior. Oxford: Wiley.

[ref96] Zeidner M. Matthews G. Roberts R. D. (2001). Slow down, you move too fast: emotional intelligence remains an “elusive” intelligence. Emotion 1, 265–275. doi: 10.1037/1528-3542.1.3.265, PMID: 12934686

[ref97] Ziegler M. Kemper C. J. Kruyen P. (2014). Short scales – five misunderstandings and ways to overcome them. J. Individ. Differ. 35, 185–189. doi: 10.1027/1614-0001/a000148

[ref98] Zumbo B. D. Kroc E. (2019). A measurement is a choice and Stevens’ scales of measurement do not help make it: a response to Chalmers. Educ. Psychol. Meas. 79, 1184–1197. doi: 10.1177/0013164419844305, PMID: 31619844 PMC6777067

